# Development and characterization of stable anaerobic thermophilic methanogenic microbiomes fermenting switchgrass at decreasing residence times

**DOI:** 10.1186/s13068-018-1238-1

**Published:** 2018-09-06

**Authors:** Xiaoyu Liang, Jason M. Whitham, Evert K. Holwerda, Xiongjun Shao, Liang Tian, Yu-Wei Wu, Vincent Lombard, Bernard Henrissat, Dawn M. Klingeman, Zamin K. Yang, Mircea Podar, Tom L. Richard, James G. Elkins, Steven D. Brown, Lee R. Lynd

**Affiliations:** 1 Thayer School of Engineering, Dartmouth College, Hanover, NH 03755 USA; 2 BioEnergy Sciences Center, Oak Ridge, TN 37830 USA; 3 Biosciences Division, Oak Ridge National Laboratory, Oak Ridge, TN 37830 USA; 4 Graduate Institute of Biomedical Informatics, College of Medical Science and Technology, Taipei Medical University, Taipei, 106 Taiwan; 5 CNRS, UMR 7257, Aix-Marseille University, 13288 Marseille, France; 6 INRA, USC 1408 AFMB, 13288 Marseille, France; 7 Department of Biological Sciences, King Abdulaziz University, Jeddah, Saudi Arabia; 8 Department of Agricultural and Biological Engineering, The Pennsylvania State University, University Park, State College, PA 16802 USA; 9 Present Address: LanzaTech, Inc., Skokie, IL 60077 USA

**Keywords:** Lignocellulose, Anaerobic, Methanogenic, Thermophilic, Solubilization, Microbial communities, Metagenomics, *Clostridium clariflavum*

## Abstract

**Background:**

Anaerobic fermentation of lignocellulose occurs in both natural and managed environments, and is an essential part of the carbon cycle as well as a promising route to sustainable production of fuels and chemicals. Lignocellulose solubilization by mixed microbiomes is important in these contexts.

**Results:**

Here, we report the development of stable switchgrass-fermenting enrichment cultures maintained at various residence times and moderately high (55 °C) temperatures. Anaerobic microbiomes derived from a digester inoculum were incubated at 55 °C and fed semi-continuously with medium containing 30 g/L mid-season harvested switchgrass to achieve residence times (RT) of 20, 10, 5, and 3.3 days. Stable, time-invariant cellulolytic methanogenic cultures with minimal accumulation of organic acids were achieved for all RTs. Fractional carbohydrate solubilization was 0.711, 0.654, 0.581 and 0.538 at RT = 20, 10, 5 and 3.3 days, respectively, and glucan solubilization was proportional to xylan solubilization at all RTs. The rate of solubilization was described well by the equation *r* = *k*(*C* − *C*_0_*f*_r_), where *C* represents the concentration of unutilized carbohydrate, *C*_0_ is the concentration of carbohydrate (cellulose and hemicellulose) entering the bioreactor and *f*_r_ is the extrapolated fraction of entering carbohydrate that is recalcitrant at infinite residence time. The 3.3 day RT is among the shortest RT reported for stable thermophilic, methanogenic digestion of a lignocellulosic feedstock. 16S rDNA phylotyping and metagenomic analyses were conducted to characterize the effect of RT on community dynamics and to infer functional roles in the switchgrass to biogas conversion to the various microbial taxa. Firmicutes were the dominant phylum, increasing in relative abundance from 54 to 96% as RT decreased. A *Clostridium clariflavum* strain with genetic markers for xylose metabolism was the most abundant lignocellulose-solubilizing bacterium. A Thermotogae (*Defluviitoga tunisiensis*) was the most abundant bacterium in switchgrass digesters at RT = 20 days but decreased in abundance at lower RTs as did multiple Chloroflexi. Synergistetes and Euryarchaeota were present at roughly constant levels over the range of RTs examined.

**Conclusions:**

A system was developed in which stable methanogenic steady-states were readily obtained with a particulate biomass feedstock, mid-season switchgrass, at laboratory (1 L) scale. Characterization of the extent and rate of carbohydrate solubilization in combination with 16S rDNA and metagenomic sequencing provides a multi-dimensional view of performance, species composition, glycoside hydrolases, and metabolic function with varying residence time. These results provide a point of reference and guidance for future studies and organism development efforts involving defined cultures.

**Electronic supplementary material:**

The online version of this article (10.1186/s13068-018-1238-1) contains supplementary material, which is available to authorized users.

## Background

Anaerobic fermentation of lignocellulose is a fundamental microbial process with central importance to the global carbon cycle, digestion of anthropogenic wastes, ruminants and wood-eating insects; it is also of interest in the context of sustainable production of heat, electricity, fuels and chemicals [1, 2]. In all these systems, solubilizing solid plant fiber is a prerequisite first step in order for components thereof to be biologically transformed, and is generally rate-limiting to performance and function [1, 3].

Rates of lignocellulose solubilization vary widely depending on the feedstock, bioreactor configuration, and operating conditions, but generally increase with increasing temperature for both pure cultures [3, 4] and consortia [5–7]. The rate of cellulose (Avicel) solubilization by pure cultures of *Ruminiclostridium thermocellum* (formerly *Clostridium thermocellum*) is proportional to cell concentration at the start of a batch culture and the remaining substrate concentration during the later stages, with specific growth rate exhibiting a maximum and variable throughout digestion [8]. Lignocellulose digestion is commonly modeled as first-order kinetics in substrate, expressed in terms of either remaining carbohydrate or biogas production [9, 10]. A transition from methanogenic to acidogenic digestion has often been observed to accompany operation at low residence times (RT) [11, 12], with previous studies generally reporting volatile fatty acids (VFAs) as the dominant products for RTs less than 5 days [13–16].

Lignocellulose-degrading microbiomes are typically dominated by Firmicutes, Bacteroidetes, and Proteobacteria [17–19]. Community analyses indicate lower diversity and higher relative abundance of cellulolytic and xylanolytic species at thermophilic temperatures [13, 20, 21]. The presence and role of members in lignocellulosic communities has often been inferred from genomic potential [17, 22–25], based upon gene expression, biochemical and activity studies of analogous enzyme systems [26–29]. Isolation and thorough characterization of microbes and enzymes are slower approaches than system-based techniques, which generate large amounts of data on many uncultured microbes and potentially novel carbohydrate-active enzymes (CAZymes) [30–33]. Enzymes containing multiple CAZyme modules with related substrate specificities have been shown to act synergistically and are more effective at degrading substrates than non-modular enzymes [3, 34, 35]. Enzyme synergy is further improved when multiple modular enzymes are docked onto a protein scaffold to form a cellulosome or are otherwise collocated [36, 37]. As more CAZymes are characterized, a greater breadth of substrate specificities are being found within enzyme families, impeding direct functional annotation [29]. Several studies have concluded that microbes with a greater diversity and number of CAZymes have higher lignocellulosic deconstruction activities than ones with less extensive inventories [38–42].

In the context of liquid biofuel production, lignocellulose solubilization by mixed microbiomes provides guidance for the development of industrial processes using defined cultures; in particular by providing a standard of comparison for pure culture studies, insights into the mechanisms operative in microbiomes, and a potential source of new biocatalysts with desirable properties [43, 44]. Motivated by these factors, we undertook to develop stable, thermophilic, lignocellulose-fermenting, methanogenic microbiomes fed at regular intervals. We report here carbohydrate solubilization, methane production, production of organic acids, and community characterization over a range of RTs. Solubilization kinetics and the relationship between cellulose and xylan solubilization were also examined.

## Methods

### Switchgrass feedstock characterization and preparation

Mid-season switchgrass (*Panicum virgatum* L., Cave in Rock) harvested in June, 2012 at Rock Springs Research Farm (Spring Mills, PA) and obtained via the Richard lab at Pennsylvania State University was used as the substrate in this study. Upon harvesting, the switchgrass was air-dried indoors for 2 weeks and subsequently stored under dry and dark conditions in mesh bags. Above-ground plants including stalks were milled to 2 mm in a ED-5 Wiley Mill (Thomas Scientific, Swedesboro, NJ), which was used for initial enrichment and inoculum preparation (described below). The 2 mm switchgrass was milled in a ZM200 centrifugal milling machine (Retsch, Haan, Germany) to a cut-off size of 0.5 mm and used for this study. All milled switchgrass passed through a 0.5 mm screen. The moisture level, chemical oxygen demand (COD), ash and carbohydrate content of the milled switchgrass (0.5 mm) is summarized in Table 1. Moisture level was analyzed by A&D MX-50 Moisture Analyzer (A&D Company, San Jose, CA). Ash content was determined by Vulcan 3-550 Muffle Furnace (DENTSPLY Ceramco, York, PA) according to the method described in [45]. COD was measured with the method described below. The total carbohydrate content of switchgrass was measured by quantitative saccharification, which is described below, and reported as glucose, xylose and arabinose.

**Table 1:** Moisture level, COD, carbohydrate and ash content of the milled switchgrass (Results are expressed as mean ± SD)

Parameters	Switchgrass value
Moisture	6.05% ± 0.3%
Total COD (g/g switchgrass)	1.22 ± 0.01
Glucose	30.6% ± 1.4%
Xylose	22.9% ± 1.0%
Arabinose	4.07% ± 0.2%
Ash	5.92% ± 0.05%

The COD, carbohydrate and ash content values are for switchgrass inclusive of 6.05% moisture

### Initial enrichment and inoculum preparation

For the initial enrichment inoculum, a digestate sample was obtained from a two-stage (aerobic then anaerobic) digester located at Vermont Technical College (http://digester.vtc.edu/, Randolph, Vermont), which was fed a mixture of farm manure and clean food waste residuals and running at mesophilic temperature. To prepare the inoculum, digestate (450 mL) was suspended in 550 mL of water with 30 g of switchgrass in a 1.5 L of bioreactor (described below in “Fermentation system” section). Effluent removal and feeding were performed daily over a period of 4 months. The inoculum reactor was fed 30 g/L of mid-season harvested switchgrass milled to 2 mm and water, with no other medium components. The residence time (RT) was 20 days for the first 112 days, and 14 days for the last 14 days. This enrichment period at 55 °C allowed a thermophilic switchgrass-fermenting community to develop and also ensured that the concentration of solids present in the original inoculum was vanishingly small by the time bioreactor studies were initiated. From this initial enrichment, three 200 mL broth liquid samples were obtained for subsequent bioreactor studies described below.

### Fermentation medium components

The following stock solutions were used: Wolfe’s modified elixir stock solution (50×) [46] contains the following chemicals in water at the indicated concentrations in gram per liter: nitrilotriacetic acid (NTA), 1.5; MgSO_4_·7H_2_O, 3.0; MnSO_4_·H_2_O, 0.5; NaCl, 1.0; FeSO_4_·7H_2_O, 0.1; Co(NO_3_)_2_·6H_2_O, 0.1; CaCl_2_·2H_2_O, 0.1; ZnSO_4_·7H_2_O, 0.1; NiCl_2_·6H_2_O, 0.05; CuSO_4_·5H_2_O, 0.01; AlK(SO_4_)_2_·12H_2_O, 0.01; H_3_BO_3_, 0.01; Na_2_MoO_4_·2H_2_O, 0.01; Na_2_WO_4_·2H_2_O, 0.01; Na_2_SeO_4_, 0.001; complete volume with Milli-Q water. Wolfe’s vitamin solution (50×) [46] contains the following chemicals in water at the indicated concentrations in milligram per liter: biotin, 2.0; folic acid, 2.0; pyridoxine hydrochloride, 10.0; thiamine·HCl, 5.0; riboflavin, 5.0; nicotinic acid, 5.0; calcium D-(+)-pantothenate, 5.0; vitamin B_12_, 0.1; *p*-aminobenzoic acid, 5.0; thioctic acid, 5.0; complete volume with Milli-Q water. Ammonium and phosphate stock solution (50×) contains 50 g/L NH_4_Cl and 25 g/L KH_2_PO_4_. Iron stock solution (1000×) contains 37 g/L FeCl_2_·4H_2_O in acidified water (500 μL of 12.1 N HCl was added to per 50 mL of water) to avoid oxidation. The solutions were either autoclaved (iron stock solution) or filter-sterilized (all other stock solutions). Switchgrass was added (without sterilization) to a concentration of 30 g/L (as-is basis with 6.05% moisture), and the pH of the medium was adjusted to pH 7.5 during each feeding event with 1 N sodium hydroxide solution.

### Fermentation system

Both the initial enrichment and subsequent bioreactor studies (R1, R2 and R3) were carried out in 1.5 L bioreactors (without sterilization) with a working volume of 1 L and operated by a Qplus multi-bioreactor system (Sartorius Stedim, Bohemia NY). The bioreactors were stirred at 280 rpm. The temperature was controlled at 55 °C by a circulating Polystat water bath system (Cole-Parmer, Vernon Hills, IL). Software accompanying the Qplus bioreactor system was used to record data for pH, base addition, temperature and stirrer speed. The pH was monitored using an InPro3253/225/Pt100 pH probe (Mettler-Toledo, OH). The pH of the growth medium fed to the reactors R1, R2 and R3 was adjusted to 7.5 at room temperature, which was sufficient to keep the reactor pH above 6.0 for all residence times. After manual adjustment of medium pH, automatic base addition in general did not occur during reported steady states. Occasional base addition took place in transients following changes in the RT.

### Fermentation start up and operation

Bioreactor studies of switchgrass-fermenting microbiomes were initiated by manually adding pre-sterilized 20 mL Wolfe’s modified elixir stock solution, 20 mL Wolfe’s vitamin solution, 20 mL ammonium and phosphate stock solution, and 1 mL iron stock solution to 30 g mid-season switchgrass prepared as above in sterile milliQ water. Effort was made to minimize contamination but the medium addition procedure was not aseptic. As an inoculum, 200 mL of broth liquid obtained from the enrichment reactor (described above) was added to a total volume of 1 L. Reactors were purged with N_2_ before and 30 min after inoculation. Reactors were operated semi-continuously by removing 100 mL of slurry (sample used for analysis) first and immediately after that adding 100 mL of medium at a frequency of 10 times per RT at equal time intervals.

**Fig. 1:**
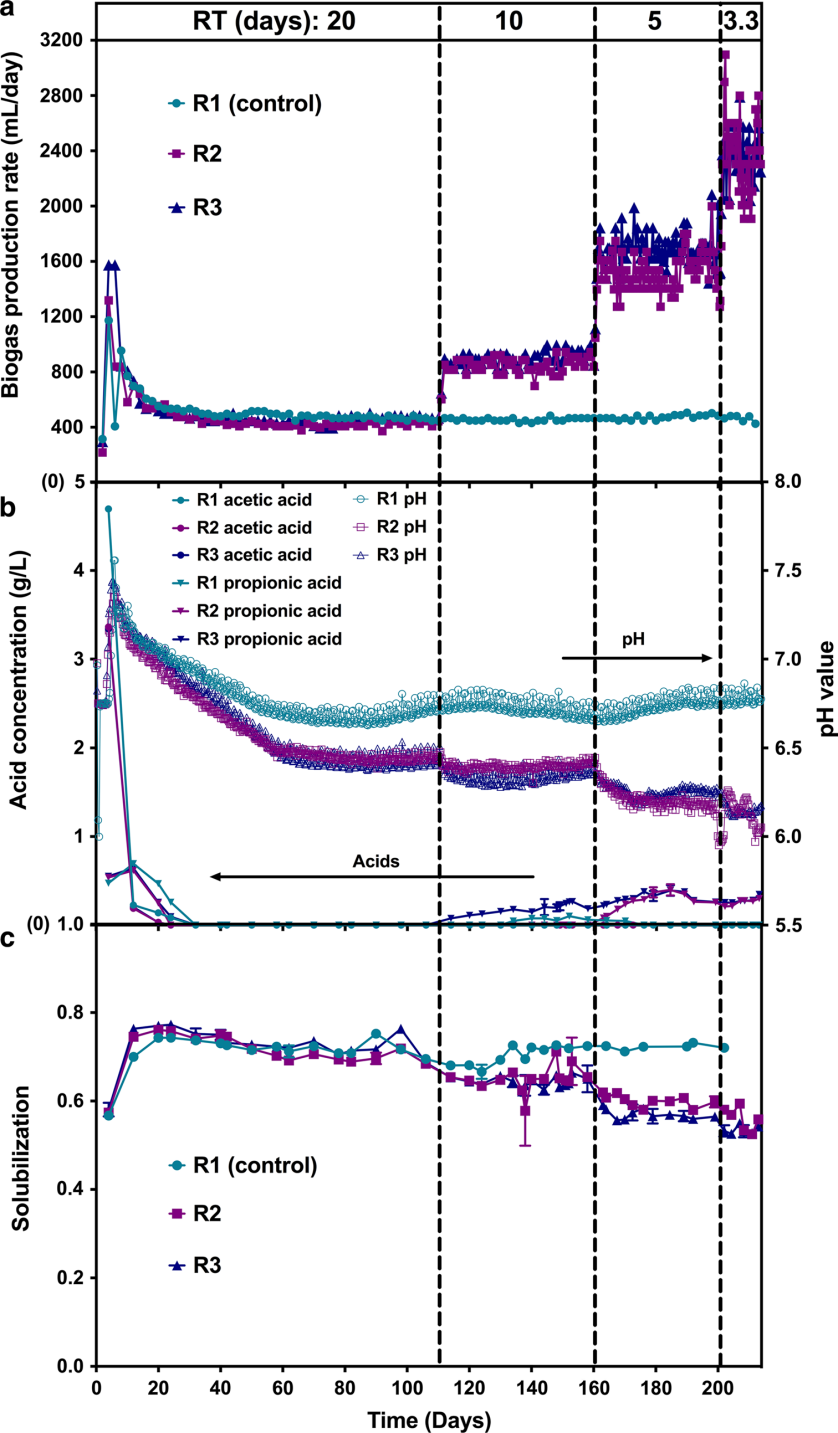
Microbiome performance shown by time-course measurements. **a** Biogas production rate (mL/day) vs. time. **b** Volatile fatty acids (VFA) concentration (g/L) and pH vs. time. **c** Fractional carbohydrate solubilization vs. time. R1 was the control reactor and maintained at residence time (RT) = 20 days throughout. R2 and R3 had decreasing RTs (20 days, 10 days, 5 days and 3.3 days) with each RT’s period indicated by dashed black lines. Biogas production was recorded once per sampling interval (1/10th RT) and normalized to daily rate assuming constant rate during per interval. pH data was automatically acquired once per 5 min and subsampled to 3–4 data points per day to be shown in figure. *FCS* data were calculated based on total carbohydrates loaded and total residual carbohydrate in each sample. Acid concentration and *FCS* results are expressed as mean ± SD

Bioreactors R1, R2, and R3 were operated for 214 days following inoculation. R1 was maintained at RT 20 days. Reactors R2 and R3 were run at RT = 20 days for 110 days, RT = 10 days for 50 days, RT = 5 days for 40 days and RT = 3.3 days for 14 days. Reactors were considered to have reached steady state after 3 RTs. From data presented in Fig. 1, stable biogas production occurred much sooner at 2 RTs (see also Additional file 1: Figure S1). For R1 and R3, slurry was withdrawn via a 50 mL pipette by removing a plug from the head plate. For R2, slurry was withdrawn by means of a 4 mm internal diameter stainless steel tube extending into the broth. Although the sampling procedure used for R2 was accompanied by less exposure of the culture to oxygen than the procedure used for R3, results were very similar for all variables monitored. In support of this statement, the maximum difference over the range of 4 residence times was tested with respect to mean fractional solubilization (0.026) and gas composition (1.6% CH_4_ and 2.4% CO_2_) (see data in Additional file 2: Table S1, Additional file 3: Table S2 and Additional file 4: Table S3). Separate control experiments for testing sampling uniformity indicated that the concentration of solids removed by both methods was equal.

### Biogas measurement

The biogas production rate was measured using a wet tip gas meter (http://wettipgasmeter.com/meters.php) filled with water acidified using either H_2_SO_4_ or HCl until pH < 2. Data were manually recorded or recorded by a data logger (HOBO Pendant Event, 64 K, Onset Computer Corporation, Pocasset, MA) at each reactor feeding. To measure the concentration of CH_4_, CO_2_, H_2_ and N_2_ gas a 0.1–0.5 mL gas sample was taken from a gas-sampling port in the gas-out line between the reactor and the gas tip meter and analyzed with a model 310 Educational Gas Chromatograph (SRI Instruments, Torrance, CA) with a thermal conductivity detector using a 1.8 m × 3 mm (6′ × 1/8″) S.S. HayeSep D packed column (SRI Instruments, Torrance, CA). Helium (20 mL/min) was used as carrier gas for CH_4_, CO_2_ and N_2_ with the column at 40 °C. Nitrogen (13.5 mL/min) was used as carrier gas for H_2_ analysis with the column at 50 °C. For all gas analyses the detector was at 150 °C.

### Fractional carbohydrate solubilization

Broth samples were centrifuged (10 min at 2800×*g*) and the pellets were freeze-dried overnight in a lyophilizer (Labconco, Kansas City, MO). Total carbohydrate content (glucan, xylan and arabinan) of the initial switchgrass material and the harvested residuals was determined by quantitative saccharification as described in [47] and adapted elsewhere [48]. The carbohydrate content of microbes is on the order of 2% (w/w) of the cell dry weight, and was considered to have a negligible contribution to the total carbohydrate content. The hydrolyzed sugars were analyzed by HPLC (Waters, Milford, MA) with an Aminex HPX-87H column (Bio-Rad, Hercules, CA) at 60 °C and detected by refractive index. HPLC eluent was 5 mM sulfuric acid with a flow rate of 0.6 mL/min. Fractional carbohydrate solubilization, *FCS*, was calculated using the following equation, with carbohydrate on a soluble monomer equivalent basis:

1 $${FCS} = \frac{{{\text{Mass carbohydrate initial}} - {\text{mass carbohydrate final}}}}{\text{Mass carbohydrate initial}} = \frac{{R_{\text{o}} \times S_{\text{o}} - C \times V }}{{R_{\text{o}} \times S_{\text{o}} }}$$

where *R*_o_ denotes the mass ratio of carbohydrate per dry solids (g monomer equivalent/g dry solids), *S*_o_ denotes the solid concentration (g/L), *C* denotes the concentration of carbohydrate in the reactor (g monomer equivalent/L), and *V* denotes the volume of the reactor. *R*_o_ and *C* are calculated for either total carbohydrate, glucan, or xylan as indicated in the text. All measurements were conducted in duplicate. Steady-state solubilization data reported in Fig. 3 are based on averages of two or more samples taken after at least three residence times following a change in residence time as detailed in Additional file 4: Table S3.

### Quantification of volatile fatty acids (VFA)

Liquid samples (filtered) were analyzed by HPLC with an Aminex HPX-87H column at 60 °C as described above for formic acid, acetic acid, propionic acid, butyric acid, iso-butyric acid and valeric acid. HPLC eluent was 5 mM sulfuric acid with a flow rate of 0.6 mL/min. All measurements were conducted in duplicate against a known standard (volatile-free acid mix standard, 46975-U SUPELCO, Sigma-Aldrich).

### Mass and electron balances

Bioreactor mass balances were calculated based on the mass flow rate of methane and CO_2_ and freeze-dried slurry leaving the reactor as a percentage of the dry weight of switchgrass added per day. CO_2_ leaving the reactor included dissolved CO_2_ calculated as described by Baskaran [49]. Bioreactor electron balances were calculated based on the mass flow rate of COD of methane and slurry leaving the reactor as a percentage of the COD of the switchgrass and liquid medium added per day. The COD of switchgrass and slurry leaving the reactor was measured using Digestion Solution for COD 20–1500 mg/L Range and Digital Reactor Block 200 (Hach Company, CO). The COD of the liquid medium was < 1% of the COD of switchgrass fed to the reactor. All measurements were conducted in duplicate.

### DNA isolation and 16S rDNA sequencing

DNA was isolated from pelleted microbial cells, without separation of residual biomass, using the PowerLyzer Powersoil DNA Isolation kit from MoBio Laboratories Inc. (Carlsbad, CA) following the manufacturer’s protocol as has previously been used to investigate methanoarchaea in environmental and anaerobic digester communities [50]. Cells were lysed using a Precellys 24 high-throughput tissue homogenizer (Bertin Technologies, Montigny-le-Bretonneux, France) at 6200 rpm for one 45-s pulse. DNA was eluted in 100 μL of water and quality/quantity was assessed by Nanodrop analysis (Thermo Scientific, Wilmington, DE) and on a 1% agarose gel.

The 16S rDNA amplicon pool was prepared according to the Lundberg et al. [51] method with the following modification [52]. For template tagging, 5 cycles of amplification were performed by denaturing the reaction at 95 °C for 1 min, annealing at 50 °C for 2 min, extension at 72 °C for 2 min and cooled down to 4 °C. The product was purified with 17 μL of Agencourt Ampure XP beads (Beckman Coulter, Brea, CA) and eluted in 21 μL of water. The primers for tagging were a mixture of 515F (5′ GTGCCAGCMGCCGCGGTAA) and 806R (5′ GGACTACHVGGGTWTCTAA) for 16S rDNA V4 region. Because the “universal” primer 515F does not effectively amplify several groups of Archaea and Bacteria, we supplemented it with modified versions that included 10% 515FArch (5′ GTGKCAGCMGCCGCGGTAA) and 3% TM7 (5′ GTGCCAGCMGCCGCGGTCA). For the second PCR step, 20 μL of purified DNA from the previous step was used in a 50 μL reaction. Purified DNA was tagged with barcoded forward and reverse PCR primers. The PCR program was 1 cycle at 95 °C for 45 s and 32 cycles at 95 °C for 15 s, annealing at 60 °C for 30 s, and 72 °C for 30 s. For sequencing, the DNA samples were pooled together and purified with Agencourt Ampure XP beads (beads to DNA, 0.7 to 1 ratio). The concentration of the 16S rDNA amplicon pool was determined by Qubit using the broad range double-stranded DNA assay (Life Technologies, Carlsbad, CA). The amplicon pool and PhiX were diluted and denatured following the manufacturer’s protocol (Illumina, San Diego, CA, Part # 15039740 Rev. D). The final sequencing concentration was 9 pM with 80% amplicon pool and 20% PhiX. Paired end sequencing (2 × 251 bp) was completed on an Illumina MiSeq instrument (San Diego, CA) with a v2 500 cycle kit.

### Read processing, OTU assignment and phylogenetic analysis

Partial 16S rDNA sequencing reads were preprocessed, operational taxonomic units (OTUs) were selected and diversity metrics calculated using the methods and pipelines described in [53]. Briefly, reads were trimmed of primers with Cutadapt (v.1.9.1) [54], and then processed using a combination of UPARSE [55] and QIIME (v.1.9.1) [56]. Quality filtering of OTUs less 0.0001% was performed in accordance with recommendations from [57], and counts were subsequently rarefied to the lowest sample OTU count, 74597. Diversity metrics were visualized with MacQIIME 1.9.1 [56] and Emperor 0.9.51 plots [58]. Data were deposited into the National Center for Biotechnology Information (NCBI) Sequence Read Archive under Bioproject PRJNA395747.

Partial 16S rDNA sequences of OTUs with greater than 1% average relative abundance were aligned with the most similar cultured species found in the non-redundant NCBI database (March 1, 2016) using MUSCLE 3.8.425 (10 iterations) [59] in Geneious 8.1.5 [60]. PhyML [61] was used to construct a maximum likelihood tree, with the following parameters: generalized time reversible (GTR) substitution model with four substitution rate categories under gamma distribution, with rates inferred from the data and with no invariable sites. The tree topology was determined using nearest neighbor interchange and optimization for topology/length/rate, the node support values were determined by bootstrapping (100 bootstraps).

### Metagenome sequencing

DNA isolated from 15 samples was sent to US Department of Energy’s Joint Genome Institute for metagenome sequencing under proposal ID 502908. DNA (100 ng) was sheared to 300 bp using the Covaris LE220 and size selected using SPRI beads (Beckman Coulter). The fragments were treated with end-repair, A-tailing, and ligation of Illumina-compatible adapters (IDT, Inc) using the KAPA-Illumina library creation kit (KAPA biosystems). qPCR was used to determine the concentration of the libraries and were sequenced on the Illumina Hiseq (San Diego, CA) using 2 × 150 nt reads.

### Metagenome assembly and annotation

BBDuk adapter trimming (https://sourceforge.net/projects/bbmap/) was used to remove known Illumina adapters; parameters used were ktrim = *r*, minlen = 40, minlenfraction = 0.6, mink = 11, tbo, tpe, *k* = 23, hdist = 1, hdist2 = 1, ftm = 5. The reads were then processed using BBDuk filtering and trimming; parameters used were maq = 8, maxns = 1, minlen = 40, minlenfraction = 0.6, *k* = 27, hdist = 1, trimq = 12, qtrim = rl. At this stage, read ends were trimmed where quality values were less than 12. Read pairs containing more than three ‘N’, or with quality scores (before trimming) averaging less than 3 over the read, or length under 51 bp after trimming, as well as reads matching Illumina artifact, spike-ins or PhiX were discarded. Remaining reads were mapped to a masked version of human HG19 with BBMap; parameters used were fast local minratio = 0.84 maxindel = 6 tipsearch = 4 bw = 18 bwr = 0.18 usemodulo printunmappedcount idtag minhits = 1, which discarded all hits over 93% identity. Trimmed, screened, paired-end Illumina reads were assembled using megahit assembler [62] using a range of Kmers. Default settings for megahit parameters were used with options “–k-list 23,43,63,83,103,123”. The entire read set output from the previously described read pre-processing step were mapped to the final assembly and coverage information generated using SEAL [63]. Pfam (db v29) and KO (db IMG.nr-2016-7-22) were assigned using HMMER 3.1b2 (February 2015) and lastal 737+ as described in [64]. CAZymes were annotated following the steps described in [17] using BLAST+ [54] and HMMER.

### Binning, sample matching, and functional analysis

Automated binning programs—MaxBin2 (v2.2.1) [65], MetaBat (v0.32.4) [66], and MyCC (v1) [67]—were evaluated with four metagenome samples prior to binning all metagenome samples. Effectiveness of binning was based on metrics provided by CheckM (v.1.0.7) [68] including average % completeness of bins, average % contamination of bins, average % strain heterogeneity of bins, and the number of bins. Default parameters of each binning program were tested. Since the default minimum contig length of MetaBat was 2500 nucleotides, MaxBin2 and MyCC were also tested with this parameter. The “superspecific” MetaBat parameter setting was also tested. Mean completeness and % of strain heterogeneity of MaxBin2, MyCC, and MetaBat were very similar (Additional file 5: Figure S2). A significant difference in contamination at *α* = 0.05 was detected by analysis of variance, and MaxBin2.2500 had the lowest average bin contamination (Additional file 5: Figure S2). Though a Tukey–Kramer test confirmed that the number of bins generated by MyCC exceeded that with MaxBin2.2500, the quality of bins was a higher priority for this study. Therefore, MaxBin2 with minimum contig length of 2500 nucleotides was used to bin all remaining metagenomes.

A customized Perl script, mapBin.pl (called MapBin for the remainder of this article), was developed to identify MaxBin2 genomic bins belonging to the same organisms but extracted from different metagenomes. Protein-coding genes were first predicted from the bins extracted from different metagenomes using FragGeneScan (v1.30) (with parameter-complete = 0-train = complete) [69]. Then predicted proteins were compared among every pair of bins from every metagenome using RAPSearch2 (v2.22) with default parameters [70]. Two bins were considered to be from the same organism if at least 70% of the protein identities were > 90%. The final output of MapBin was a list indicating the most similar bins from one sample to another along with their proportion of highly similar protein matches. MapBin is available at https://sourceforge.net/projects/mapbin/. Simulated datasets described in [71] were used to test the effectiveness of MapBin, which successfully mapped three 20× coverage bins against 80× coverage bins as well as 30 low and medium complexity bins to high-complexity bins. The genome mapping accuracy was 100% (i.e., all mapped genomes were found to belong to the same organism). After extracting the completeness and contamination ratios using CheckM, we found that most of the bins with less than 10% contamination and greater than 50% completeness can be mapped very well (Additional file 6: Figure S3). Because MapBin also matched bins with much higher contamination and much lower completeness, we used thresholds of less than 20% contamination and greater than 40% completeness to reduce false-positive rates when evaluating genomic potential.

Pairwise comparisons of genomes binned from the switchgrass anaerobic digester metagenomes meeting the 70% protein match and 90% identity MapBin thresholds were combined into a single list. Pairwise comparisons where one or both bins had greater than 20% contamination or less than 40% completeness were removed in accordance with thresholds used in [68, 71], respectively. Remaining pairwise comparisons were imported into Cytoscape (v.3.4.0). Bins connected by less than three edges were separated, and only networks containing bins from more than three metagenomes were further analyzed. Genetic potential was evaluated with the remaining bins using genetic markers of interest including carbohydrate-active enzymes (CAZymes), markers with KEGG orthology (KOs) for xylose metabolism, and protein family (pfams) markers for syntrophy and methanogenesis, which were derived from [72] and MetaCyc.org [73] (Additional file 7: Table S4). Organisms were considered to encode a genetic marker if the majority of matching bins contained the marker. Taxonomy was assigned using CheckM [68], and manually updated if the average nucleotide identity % calculated with both BLAST+ and MUMmer using the JSpecies webserver were greater than 95% [74].

## Results

### Microbiome establishment and performance at decreasing residence times

Anaerobic microbiomes derived from a digester inoculum were incubated at 55 °C and fed semi-continuously with medium containing 30 g/L mid-season harvested switchgrass to achieve residence times (RT) of 20, 10, 5, and 3.3 days (Additional file 8: Table S5). Steady-state data for these 4 RTs studied are presented in Additional file 4: Table S3, Additional file 1: Figure S1 and Additional file 9: Figure S4, including biogas production rate and composition, fractional carbohydrate solubilization, volatile fatty acids (VFA) concentration, pH and mass and electron balances. Mass and electron balances (Additional file 4: Table S3 and Additional file 9: Figure S4) closed rather well between 84 and 106%. Gas production increased with decreasing RT (Fig. 1a and Additional file 4: Table S3). The gas composition (Additional file 4: Table S3 and Additional file 1: Figure S1) was primarily methane and CO_2_. Progressing from RT = 20 days to 3.3 days, methane concentration decreased slightly from 50.5 ± 1.0 to 46.7 ± 0.7% and CO_2_ increased from 42.6 ± 2.2 to 45.9 ± 1.5%. Minor amounts (< 10%) of N_2_ were detected likely because of the feeding and sampling process. A peak comigrating with H_2_ was detected, but was ≤ 0.1% of the fermentation gas mixture (100-fold lower than a 10% H_2_ standard).

Time courses for production of VFA and pH are presented in Fig. 1b. VFA concentrations briefly exceeded 3 g/L following inoculation, but were not detected after 30 days at RT = 20 days for all three reactors. Total VFAs were detectable at shorter RTs but never exceeded 0.5 g/L. Propionic acid was the main VFA accumulated, with acetic acid also observed; peaks with retention times corresponding to butyric, isobutyric and valeric acids were observed occasionally at concentrations near detection limits of 0.1 g/L (see Additional file 4: Table S3). At RT = 20 days, pH stabilized at about 6.7 for R1, and 6.45 for R2 and R3. With decreasing RT, pH values for R2 and R3 were very similar, declining during cultivation as follows: 6.4–6.5 at RT = 20 days, 6.3–6.4 at RT = 10 days, 6.2 at RT = 5 days, and 6–6.2 at RT = 3.3 days (see Additional file 4: Table S3).

**Fig. 2:**
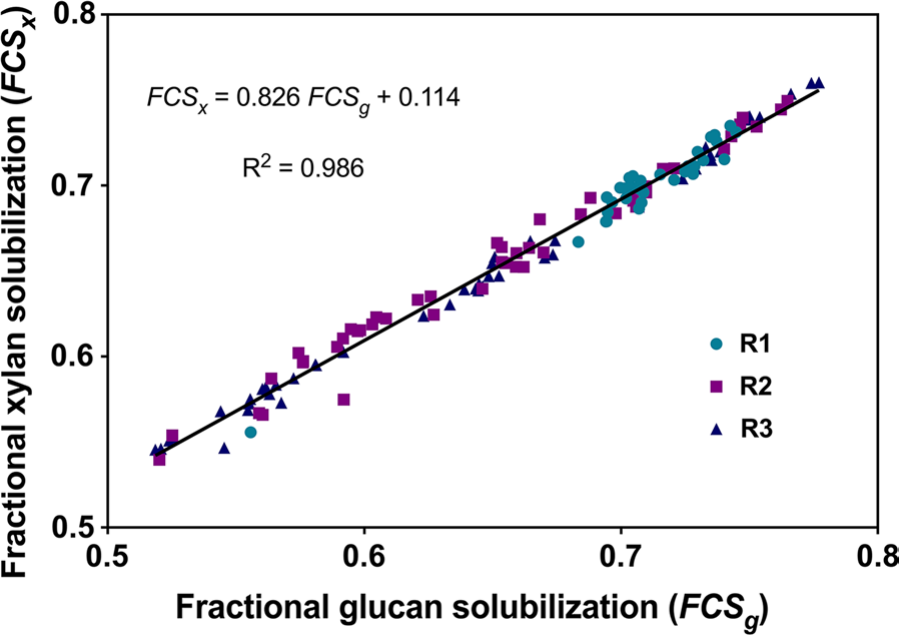
Comparative solubilization of xylan and glucan. Data are for the same samples reported in Fig. 1. Best fit linear regression curve is shown as solid black line

Carbohydrate solubilization reached steady state at each of the four RTs studied after a transient period of three RTs (Fig. 1c). Average steady-state fractional carbohydrate solubilization were 0.711 ± 0.021 at RT = 20 days (based on R1, R2 and R3), 0.654 ± 0.023 at RT = 10 days (R2 and R3), 0.581 ± 0.017 at RT = 5 days (R2 and R3), and 0.538 ± 0.015 at RT = 3.3 days (R2 and R3) as documented in Additional file 4: Table S3. Each of these mean values was significantly different from the values at the nearest residence times at *p* = 0.05. Fractional xylan solubilization, *FCS*_*x*_, is plotted as a function of glucan solubilization, *FCS*_*g*_, for all reactors and all RTs in Fig. 2, and is described well by the linear equation ${FCS}_{x} \, = \,0.826*{FCS}_{g} \, + \,0.114$.

**Fig. 3:**
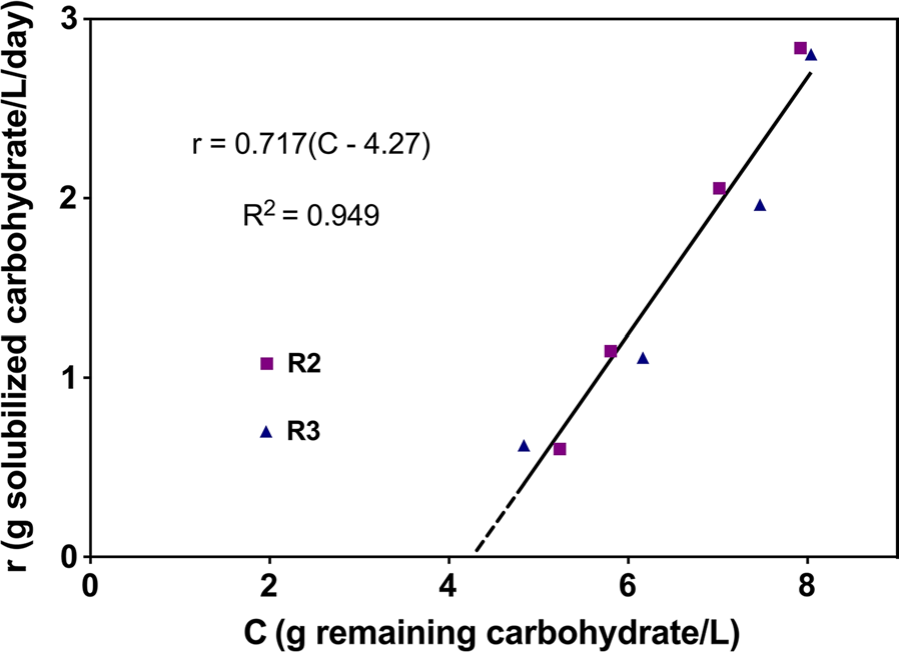
Rate of total carbohydrate solubilization *r* (g solubilized carbohydrate/L/day) vs. concentration of unutilized carbohydrate C (g remaining carbohydrate/L). Mean of steady-state solubilization data of each residence time (RT) of R2 and R3 (shown in Additional file 4: Table S3) was used to calculate *C* and *r*. Linear regression was performed and fitting curve was shown as solid black line in the figure

Figure 3 plots the rate of carbohydrate solubilization, *r* (g/L day), as a function of the concentration of unutilized carbohydrate, *C*, both on a sugar monomer equivalent basis. The rate is observed to increase linearly with increasing concentration but to have a non-zero concentration intercept at zero rate. This behavior is consistent with kinetics being first order in accessible substrate, described by

2 $$r = k\left( {C{-}C_{0} f_{\text{r}} } \right)$$

where *k* is a rate constant, *C*_0_ is the concentration of carbohydrate (cellulose and hemicellulose) entering the bioreactor (*C*_0_ = 17.3 g/L) and *f*_r_ is the extrapolated fraction of entering carbohydrate that is recalcitrant at infinite residence time. Using data from both R2 and R3, the best fit value of *k* was 0.717 ± 0.068 day^−1^. The best fit value of *f*_r_ was 24.7 ± 3.6%.

### Microbiome composition in switchgrass digesters with decreasing residence times

**Fig. 4:**
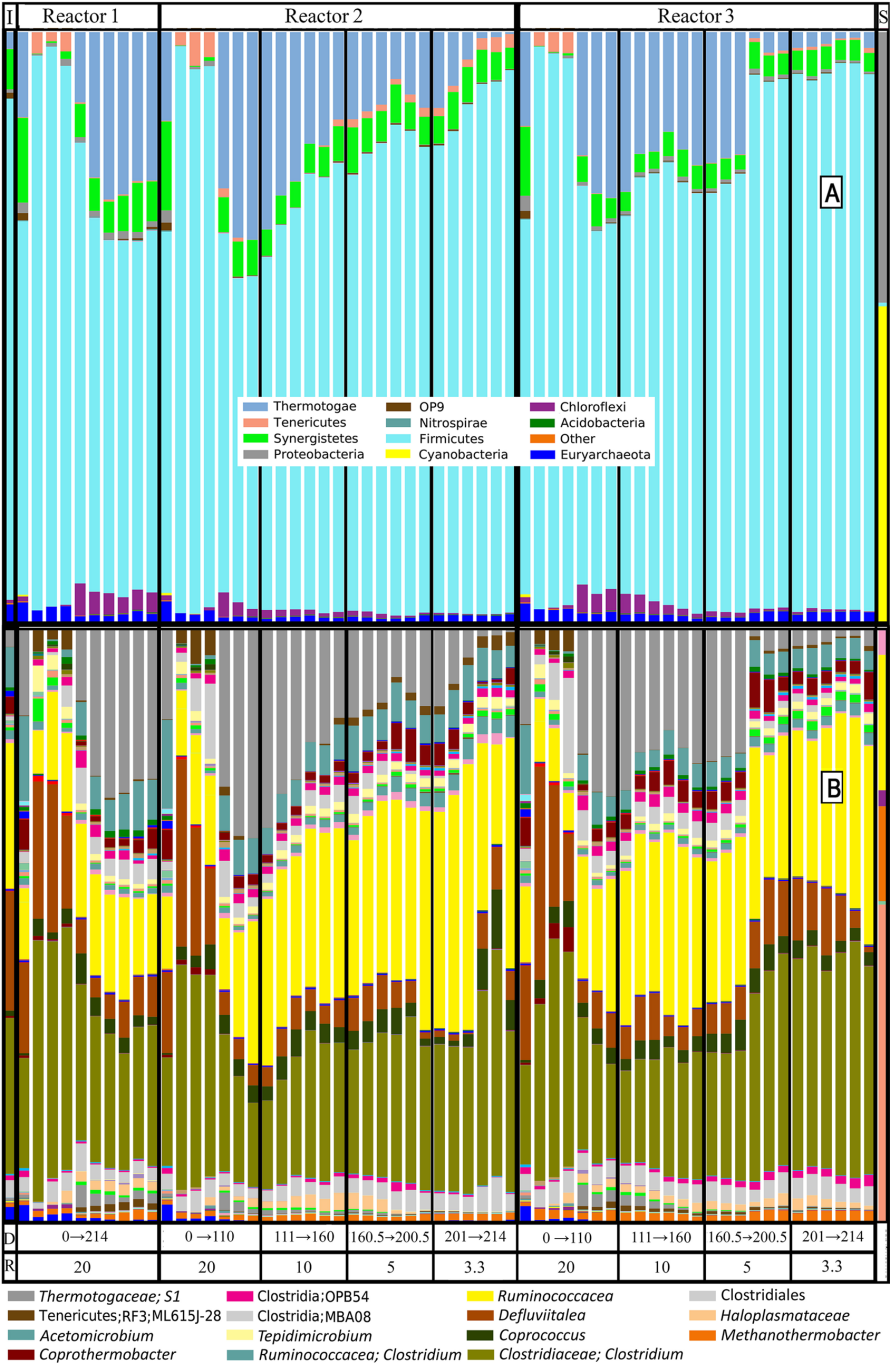
Relative abundance of the bacterial and archaeal partial 16S rDNA genes with phylum (**a**) and taxon level resolution as low as genus (**b**) in switchgrass digesters. Fermentation periods are divided by days (D) and residence time (*R*). The left and right columns are the inoculum (*I*) and substrate (*S*) profiles. Only taxa with average relative abundances of greater than 1% are indicated in the legend of **b**. Steady state with respect to solubilization and gas composition was in general observed at times following change of the feed rate ≥ 3 residence times as described elsewhere. Taxonomic profiles are presented both before and after this 3 residence time threshold. The number of taxonomic profiles made after this threshold for reactors R2 and R3, corresponding to the data to the right for each RT, were as follows: 3 for RT = 20 days, 2 for RT = 10 days, 3 for RT = 5 days, 1 for RT = 3.3 days

Bacteria and archaea in switchgrass digesters were monitored by the relative abundance of amplified partial 16S rDNA genes. Figure 4 shows the phylum and taxon-level dynamics of bacteria and archaea with greater than 1% average abundance, and a PCoA graph provided in the supplemental materials shows sample-level changes by weighted UniFrac (Additional file 10: Figure S5). At all levels, the population dynamics were very similar in experimental reactors R2 and R3. Firmicutes were the dominant phylum, increasing in inferred relative abundance from 54–96% as RT decreased. Thermotogae and Chloroflexi decreased with lower RT. Indeed, the most striking feature of the population dynamics was the decrease in a *Thermotogaceae* as RT was decreased from 20 to 3.3 days. This decline was more gradual in reactor 2, but in reactor 3, an abrupt drop in abundance was detected between 162 and 180 days of fermentation time when the RT was 5 days. *Haloplasmataceae* was more persistent than the other Chloroflexi in reactors 1 and 3, but the highest relative abundance was observed in reactor 2 until the population declined sharply at RT = 5 days. Rapid decline of populations can occur when the dilution rate is greater than the growth rate, a phenomena termed washout [75]. Synergistetes (dominated by *Acetomicrobium*) and Euryarchaeota (dominated by *Methanothermobacter*) were relatively stable in all reactors. An organism from the RF3 clade of Tenericutes was also present in most samples with a higher relative abundance in reactor 2 than 1 or 3. Uncultured representatives of the RF3 clade are like Mollicutes in that they ferment sugars into organic acids, but they also have the genetic potential to produce H_2_ from resulting reducing equivalents [76]. Proteobacteria, OP9, Nitrospirae, Gemmatimonadetes, Cyanobacteria, Acidobacteria, and other phyla each represented less than 1% of the average relative abundance of samples.

To gain a better understanding of the metabolic role of the most abundant species in the reactors, a phylogenetic tree was created (Additional file 11: Figure S6). Six of the operational taxonomic unit (OTU) sequences matched the partial 16S rDNA of previously isolated species. Isolates include thermophilic cellulose and hemicellulose fermenters, *Clostridium clariflavum* [77, 78] and *Defluviitoga tunisiensis* (*Thermotogaceae* in Fig. 4); a cellobiose and arabinose fermenter, *Clostridium caenicola* [79]; an *Acetomicrobium* (synergistetes in Fig. 4a) that ferments starch, glycerol, some monosaccharides and organic acids [80]; *C. proteolyticus*, previously shown to have high proteolytic activity in an office paper digester [81, 82], and a thermophilic hydrogenotrophic methanogen, either in the genus *Methanothermobacter* or *Methanobacterium*. Many of the other OTUs are related to bacteria known to ferment cellulose, cellobiose, xylan, xylose or a combination of these as well as other saccharides released from plant biomass degradation into products including organic acids (acetate, propionate, and butyrate), H_2_ and CO_2_. One was also related to *Clostridium ultunense*, a syntrophic acetate oxidizer [83].

### Carbohydrate solubilization genetic potential of lignocellulose microbiome members

**Fig. 5:**
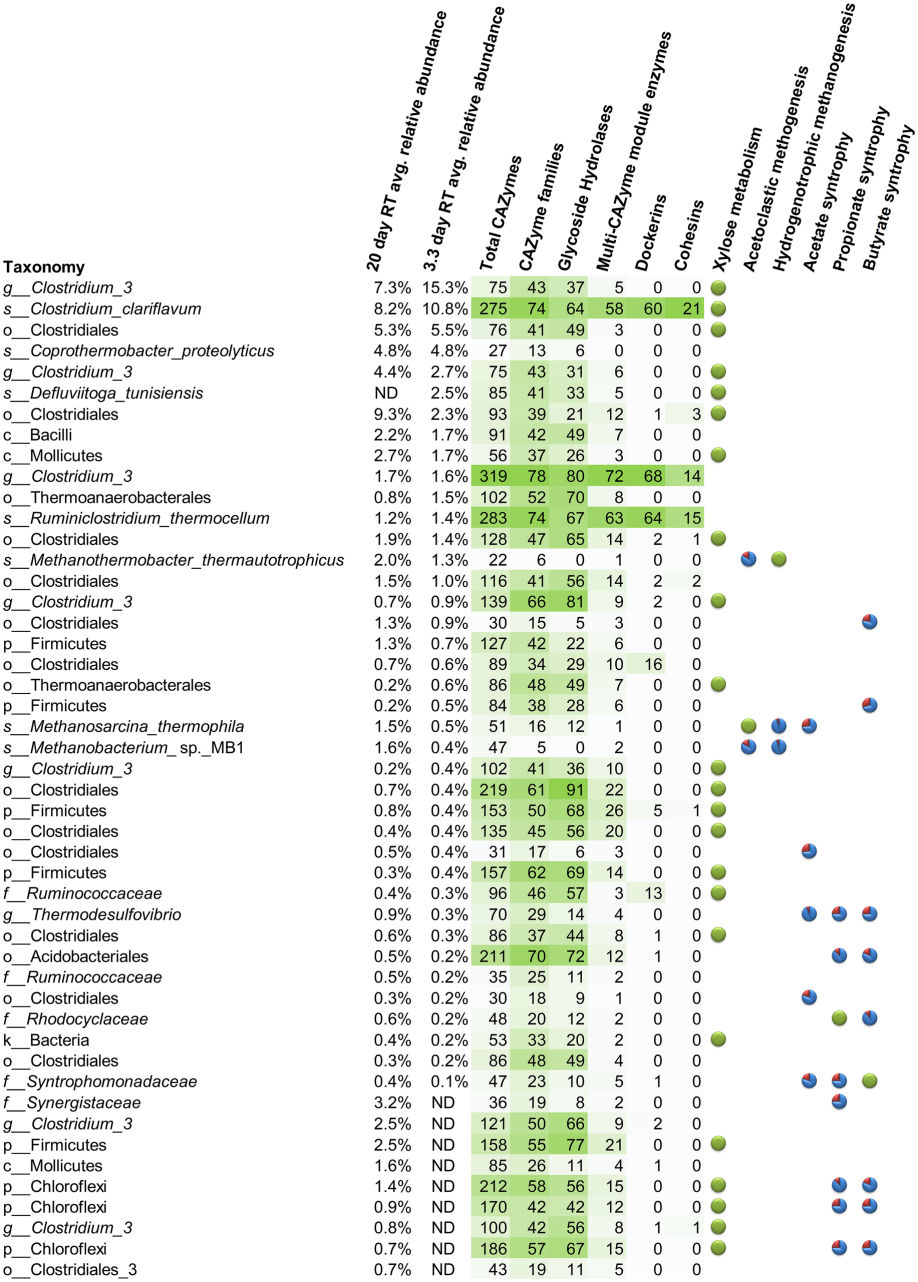
Switchgrass digester microbial community with CAZymes profiles and key metabolic pathways. Each microbe listed was found in at least four of fifteen metagenome samples taken from three reactors operating at residence times (RT) of 20, 10, 5, and 3.3 days. Taxonomy was assigned using CheckM; species were manually assigned if its average nucleotide identity was greater than 95% the type strain genome. Microbes were sorted by average relative abundance at the 3.3-day RT. Average relative abundance was not determined (ND) for microbes whose metagenome bins were less than 40% complete or had greater than 20% contamination. Darker green colors indicate higher counts of CAZyme markers. Marker sets used to evaluate metabolic potential were taken from MetaCyc.org and Worm et al. [72]. Complete marker sets are indicated by green pie graphs, while incomplete marker sets with greater than 70% completeness are indicated by red and blue pie graphs, where red indicates the percentage of missing markers

Metagenomes were generated from samples taken from all three reactors at all RTs (15 metagenome total with an average of 226,652 sequences, 257,340,992 nucleotides, and 409,586 genes) and genomes were then binned to characterize the genetic potential of members of the switchgrass digester microbiome. Two hundred and seventy-five bins (35.5%) were greater than 95% complete. Only 77 (9.9%) were less than 40% complete and only 118 (15.2%) contained greater than 20% contamination. Addition statistics for the metagenome assemblies and bins are provided in Additional file 12: Table S6. Figure 5 shows the average relative abundance of microbes in switchgrass digesters with average relative abundances greater than 0.1% and present in at least four of the fifteen metagenome samples. The abundance and taxonomy identified by metagenomics matched well with 16S rDNA data, although there were understandable differences due to species-specific variation in scaffold retention and 16S rDNA copy number, and marker-set verses single-gene-based taxonomic assignments, and inherent limitations for both methods. For example, metagenomics indicated that a cluster III *Clostridium* was the most abundant microbe at RT = 3.3 days with an average relative abundance of 15.3% and had the largest increase in relative abundance, up from 7.3% at RT = 20 days. Similarly, 16S rDNA dynamics show a *Clostridium* approximately doubling when comparing day 6 or 110 (RT = 20) to day 214 (Fig. 4). The cluster III phylogenic group contains primarily cellulose-degrading *Clostridium* [78, 79], and members of this cluster have been observed to have higher growth rates than 3.3 days when grown in pure culture [3].

We quantified the total number of CAZymes for each member of the switchgrass digester microbiome (Fig. 5). CAZymes were further partitioned into categories: CAZyme families (to show diversity of functionality), quantity of glycoside hydrolases (since GHs are responsible for hydrolyzing the glycosidic bond of carbohydrates), quantity of modular CAZymes (because ancillary modules are often synergistic), and quantity of dockerins and cohesins (for detection of cellulosomes). Full list of modular CAZymes and individual modules including glycoside hydrolase, glycosyltransferase, polysaccharide lyases (which cleave glycoside linkages), and carbohydrate esterases detected in the metagenomes of each microbe are provided in Additional file 13: Table S7. *C. clariflavum*, a cluster III *Clostridium* (different from the most abundant organism in the switchgrass digesters at RT = 3.3 days), and *R. thermocellum* tended to dominate all of these categories (Fig. 5). Besides being the only cellulosomal bacteria in the community, they were also the only bacteria that encoded GH48 (Additional file 13: Table S7) which has been found to be critical for crystalline cellulose degradation in several bacteria [84]. *C. clariflavum* was approximately 7 times more abundant than the other two bacteria. The genome of the cellulosomal cluster III *Clostridium* had an average nucleotide identity of 75% for approximately 40% of the *R. thermocellum* ATCC27405 and DSM1313 genomes (BLAST+ method). Several of the modular enzymes of the cluster III *Clostridium* were similar in module arrangement and nucleotide identity to those encoded in *R. thermocellum*, but some were different including one that appeared to encode a novel xylanase with the modular arrangement GH11-CBM6-GH10-CBM6-DOC (Additional file 13: Table S7). We also detected one organism from the order Clostridiales and one from the family *Ruminococcaceae* which had 16 and 13 dockerins, respectively, but no cohesins, an observation that has previously been reported in other studies [17, 85].

### Metabolic features of lignocellulose microbiome members

In addition to cellulose utilization, the metabolic guild structure of the community was examined by searching bins for conserved markers and pathways involved in anaerobic digestion of organic matter to methane. Xylose catabolism, methanogenesis (acetoclastic and hydrogenotrophic), and syntrophic pathways for acetate, propionate and butyrate utilization were assessed (Fig. 5). Bins containing pfam domains corresponding to xylose isomerase (pfam01261) and xylulokinase (pfam00370, pfam02782) were widespread throughout the community and notably, in the most abundant species including *Clostridium clariflavum*. Strains of *C. clariflavum* have been shown to utilize both hexose and pentose sugars, a feature that distinguishes the organism from *R. thermocellum* [77]. While saccharolytic, fermentative bacteria dominated the microbiome, short chain fatty acids did not accumulate at the 20-day RT and remained below 1 g/L during the 10, 5 and 3.3-day RTs (Fig. 1) suggesting obligate and/or facultative syntrophic interactions were enabled by the presence of methanogens. In thermophilic systems, acetate removal via syntrophic oxidation has been observed to be favored over uptake by acetoclastic methanogens [86] and sequences associated most closely with *M. thermautotrophicus* showed the highest abundance in both the 20-day and 3.3-day RTs. Acetate oxidation occurs via the Wood-Ljungdahl (reductive acetyl-CoA) pathway operating in the reverse direction [72]. Therefore, pfam domains representing the pathway including pfam01268 for formyltetrahydrofolate synthetase, an essential function and marker gene [87], were used to query bins for the potential for acetate oxidation. Four bacteria genomes contained near-complete pathways including pfam01268 which were associated with *Thermodesulfovibrio* spp. and *Syntrophomonadaceae* (Fig. 5). Evidence for syntrophic propionate utilization was more abundant, especially for the 20-day RT, as indicated by the presence of genes with pfam domains that are included in the methylmalonyl-CoA pathway and periplasmic formate dehydrogenase which are key functions for propionate oxidation in *Syntrophobacter fumaroxidans* [72]. Reducing the RT to 3.3 days apparently washed out a portion on the predicted propionate-dependent syntrophs, especially organisms belonging to the phylum Chloroflexi [88]. Likewise, potential butyrate-dependent syntrophic organisms represented a minor, yet diverse component of the microbiome. Based on comparative genomic and functional proteomic studies, the essential genes/enzymes for butyrate oxidation in *Syntrophomonas wolfei* have been described [72, 89]. The binned genomes were searched for conserved pfam domains involved in butyrate and formate metabolism with organisms associated with *Syntrophomonadaceae* displaying a full set of predicted conserved genes necessary for syntrophic growth. Genomic bins associated with *Rhodocyclaceae* and *Thermodesulfovibrio* spp. also contained extracytoplasmic FDH and FDH accessory proteins (pfam04216) which are indicative of butyrate syntrophy. A full list of pfams identified for each microbe are provided in Additional file 7: Table S4.

## Discussion

Stable, thermophilic, lignocellulose-fermenting microbiomes were established at residence times (RT) from 20 to 3.3 days. Consistent with the objective of establishing a well-controlled system for studying lignocellulose solubilization, we developed a feeding protocol that involved replacing one-tenth of the reactor volume at regular time intervals with a consistent lignocellulose feedstock. Many other studies use different feeding protocols as determined by their aims and circumstances—including batch, fully continuous, various lignocellulosic substrates and water contents, and time-varying substrates (e.g., any actual waste). As frequently noted in the literature [90, 91], inter-study comparisons and generalized conclusions are challenging because of the large number of operational variables that impact the composition and performance of anaerobic digesters and similar systems. Acknowledging these factors, the first-order rate constant we observed for carbohydrate solubilization (0.53 day^−1^) is larger, and 3.3-day RT shorter than most reports in the literature for methanogenic digestion of lignocellulosic feedstocks [9, 10].

Anaerobic microbiomes fed mid-season switchgrass reached steady-state at each of the four RTs examined as indicated by biogas production and composition, pH, organic acid levels, and fractional carbohydrate solubilization. As compared to the intermittent feeding strategy used in our studies, data interpretation is somewhat more straight forward for reactors fed in a fully continuous manner. However, fully continuous feeding becomes infeasible at high solid concentration, theoretical frameworks for considering intermittently fed reactors are available, and such frameworks suggest that differences between fully continuous and intermittent feeding are not large as long as the fraction of culture volume replaced per feeding is relatively small [92].

Most reports of thermophilic, anaerobic, methanogenic digestion of lignocellulose and related materials are at RT > 3.3 days as observed in our study. For example, Vanwonterghem et al. [13] observed that over 70% of solubilized alpha cellulose was converted to VFAs at 55 °C and RT = 4 days. The shortest RT reported by Yilmaz et al. [93] for digestion of papermill wastewater at 55 °C was 6 days. Kim et al. [15] in 2006 digesting food waste at 55 °C observed reduced methane yield and had trouble maintaining stability as RT was reduced from 10 to 8 days. Liu et al. [14] also reported reduced methane yield for digestion of swine manure and corn stover at 50 °C when RT was lowered from 10 to 5 days. We speculate that our observation of stable methanogenesis at short residence times may be due to the well-controlled and frequent feeding regime employed.

As reported by Van Soest [94] and Richard [95], kinetics were observed to be first order for the remaining accessible carbohydrate. The inferred concentration of recalcitrant carbohydrate, corresponding to the zero-rate intercept in Fig. 3, was 24.7% of the carbohydrate contained in the substrate prior to fermentation. We expect that fully senescent switchgrass would have a yet higher fraction of recalcitrant carbohydrate compared to mid-season switchgrass as has been observed in pure culture studies with *R. thermocellum* [96, 97]. Modeling the rate of lignocellulose solubilization as first order in carbohydrate is common in studies of both anaerobic digesters and pure cultures [3, 10]. Our results suggest that accounting for recalcitrant carbohydrate can be a useful refinement of descriptive rate laws for lignocellulose solubilization, and that the fraction of recalcitrant carbohydrate may be an informative comparative metric for various microbial systems and feedstocks. Glucan and xylan solubilization were observed to be linearly correlated, consistent with the results of Paye et al. [4].

The three most abundant cellulolytic microorganisms were *C. clariflavum*, *R. thermocellum*, and an uncultured cluster III *Clostridium*. *C. clariflavum* was approximately seven times more prevalent than the other two predicted cellulosomal bacteria at all RTs, and differed in that it contained markers for xylose metabolism (Fig. 5). All three were found to encode similar overall numbers of CAZymes, CAZyme families, GHs, and modular enzymes as well as dockerin and cohesin modules, indicative of cellulosomes (Fig. 5). Thus, the number, diversity, and architecture of CAZymes were not a particularly good predictor of quantitative abundance.

*Defluviitoga tunisiensis* had the most dramatic population shift of any species in switchgrass digesters when RT was decreased from 20 to 3.3 days (Fig. 4). At the end of the incubation period with RT = 20 days, the relative abundance for *D. tunisiensis* exceeded that of two highly abundant *Clostridium* species, an uncultured cluster III *Clostridium* and *C. clariflavum* (Fig. 4). However, the average relative abundance of *D. tunisiensis* decreased to 2.5% at RT = 3.3 days (Figs. 4, 5), while these two *Clostridium* species increased in abundance to 15.3% and 10.8%, respectively. Separate previous studies have indicated a high abundance of Thermotogae in thermophilic biogas reactors operating at high RTs of 30 days [88] and 19.8 days [98] and low abundance of Thermotogae in biogas reactors operating at RTs of 4 and 3 days [12] and 1 day [99]. To our knowledge, this study is the first to capture a dynamic shift in relative abundance of a Thermotogae. *D. tunisiensis* is a primary fermenter capable of growing on microcrystalline cellulose [100], but the genome sequence of the L3 strain only encoded a GH5 cellulase [101]. The *D. tunisiensis* strain identified in this study encoded a GH5 cellulase but did not encode a GH48 cellulase (Additional file 13: Table S7), which was previously found to be an absolute requirement for pure culture crystalline cellulose hydrolysis in the genus *Caldicellulosiruptor* [102]. The relationship between certain specific CAZyme markers such as GH48, thought to be indicative of strong cellulolytic activity, other metabolic markers (e.g., xylose utilization) and to the success individual members and the overall lignocellulosic microbiome requires further quantitative study.

Genomic science opens unprecedented windows into the composition and function of microbiomes, including but not limited to microbiomes based on lignocellulosic substrates. In the context of gaining insights into lignocellulose solubilization based on microbiome studies, we see a need and opportunity for hypothesis testing based on melding bioinformatic and comparative solubilization data obtained under controlled conditions and in particular with defined microbial communities. It is also important that community standards are developed to allow microbiome comparisons between different systems and for future more detailed mechanistic insights. There is also a place for cultured lignocellulosic microbiome representatives complete with genome sequences to test certain hypotheses, such as described recently for the Hungate 1000 project which is a catalogue of reference genomes from the rumen microbiome [103]. We hope that the methods, systems, and genomic resources and results presented here will provide a foundation for such studies.

## Conclusions

In this study, we established stable, anaerobic, switchgrass-fermenting, methanogenic enrichment cultures at various residence times at 55 °C. Minimal accumulation of organic acids was observed for all RTs. Fractional carbohydrate solubilization was 0.711, 0.654, 0.581 and 0.538 at RT = 20, 10, 5 and 3.3 days, respectively. The 3.3 day RT is among the shortest RT reported for stable thermophilic, methanogenic digestion of a lignocellulosic feedstock. Features of the microbiomes were documented, including descriptive rate laws, and relative solubilization of cellulose and hemicellulose. 16S rDNA phylotyping and metagenomic analyses were conducted to infer functional roles in the switchgrass to biogas conversion to the various microbial taxa and to characterize the microbial communities present at various residence times. *C. clariflavum*, *R. thermocellum*, and an uncultured cluster III *Clostridium* were the most abundant putative cellulolytic microbes present. Although these three microbes manifested similar numbers, diversities, and architectures of CAZymes, *C. clariflavum* was the most abundant by sevenfold.

## Additional files

**Additional file 1: Figure S1.** Biogas composition vs. time. R1 was the control reactor and maintained at residence time (RT) = 20 days throughout. R2 and R3 had decreasing RTs (20 days, 10 days, 5 days and 3.3 days) with each RT’s period indicated by solid black lines. Contents of CH_4_ and CO_2_ were expressed on fractional basis.

**Additional file 2: Table S1.** ANOVA of effects of residence time (RT) and anaerobic/aerobic sampling method on total carbohydrate solubilization.

**Additional file 3: Table S2.** ANOVA of effects of residence time (RT) and anaerobic/aerobic sampling method on CH_4_ content.

**Additional file 4: Table S3.** Steady State Data Summary. Results are expressed as mean ± SD.

**Additional file 5: Figure S2.** Comparison of statistics generated from four metagenomes from reactor R3 that were binned with MaxBin2, MyCC, and MetaBat programs. Default parameters were tested. Since the default minimum contig length of MetaBat was 2500 nucleotides, MaxBin and MyCC were also tested with this parameter. The “superspecific” MetaBat parameter settings were also tested. Error bars indicate the standard deviation of averaged binning statistics of the four metagenomes. Significantly different means were identified by ANOVA at an α = 0.05 for the % contamination and number of bins. However, a Tukey–Kramer post hoc test only identified a significant difference in the mean number of bins for MyCC compared to MaxBin2.2500.

**Additional file 6: Figure S3.** MapBin. Plot indicating the completeness and contamination distribution of bins that can or cannot be mapped. X-axis indicates completeness ratio (%) while Y-axis means contamination ratio (%). Black dots are bins that can be mapped; gray ones are bins that cannot.

**Additional file 7: Table S4.** Genetic markers of interest including carbohydrate-active enzymes (CAZymes), markers with KEGG orthology (KOs) for xylose metabolism, and protein families (pfams) markers for syntrophy and methanogenesis, which were derived from [72] and MetaCyc.org.

**Additional file 8: Table S5.** Reactor operating length and steady state length at each residence time (RT). R1 was the control reactor always running at RT = 20 days and R2 & R3 had decreasing RTs.

**Additional file 9: Figure S4.** **a**. COD recovery vs. time; **b.** Mass recovery vs. time. R1 was the control reactor and maintained at residence time (RT) = 20 days throughout. R2 and R3 had decreasing RTs (20 days, 10 days, 5 days and 3.3 days) with each RT’s period indicated by solid black lines. Results were expressed as mean ± SD.

**Additional file 10: Figure S5.** PCoA plot of sample phylogenetic distance. Phylogenetic distance of samples represented by weighted UniFrac values. Residence time (RT) is represented by different colors. Reactor 1 (A1) samples are twice the size of samples for reactors 2 (A2) and 3 (A3) reactors. Samples are labeled with [reactor]_[day].

**Additional file 11: Figure S6.** Maximum likelihood phylogenetic tree of the most abundant operational taxonomic units in the switchgrass digesters (bold numbers), which when summed together account for greater than 80% of the average relative abundance. Numbers given to OTUs only allude to the abundance of the OTUs in one of the 61 switchgrass digester samples. Numbers adjacent to branches show bootstrap supports that were greater than 50%. The scale bar and numbers below the branch-breaks indicate the number of nucleotide substitutions per position in the V4 region of the 16S rDNA gene sequences.

**Additional file 12: Table S6.** Addition statistics for the metagenome assemblies and bins.

**Additional file 13: Table S7.** Total CAZyme domain counts normalized by number of matching bins for a species and modular CAZymes detected in the matching bins for each species. Taxon are ordered as seen in Fig. 5.

## Abbreviations

CAZymes: carbohydrate-active enzymes
COD: chemical oxygen demand
GTR: generalized time reversible
ND: not determined
NTA: nitrilotriacetic acid
OTU: operational taxonomic unit
RT: residence time
VFA: volatile fatty acids
