# Supplementary figures and images for: Development and characterization of stable anaerobic thermophilic methanogenic microbiomes fermenting switchgrass at decreasing residence times
Source: Biotechnol Biofuels. 2018 Sep 6;11:243. doi: 10.1186/s13068-018-1238-1 (PMC6126044; doi:10.1186/s13068-018-1238-1)

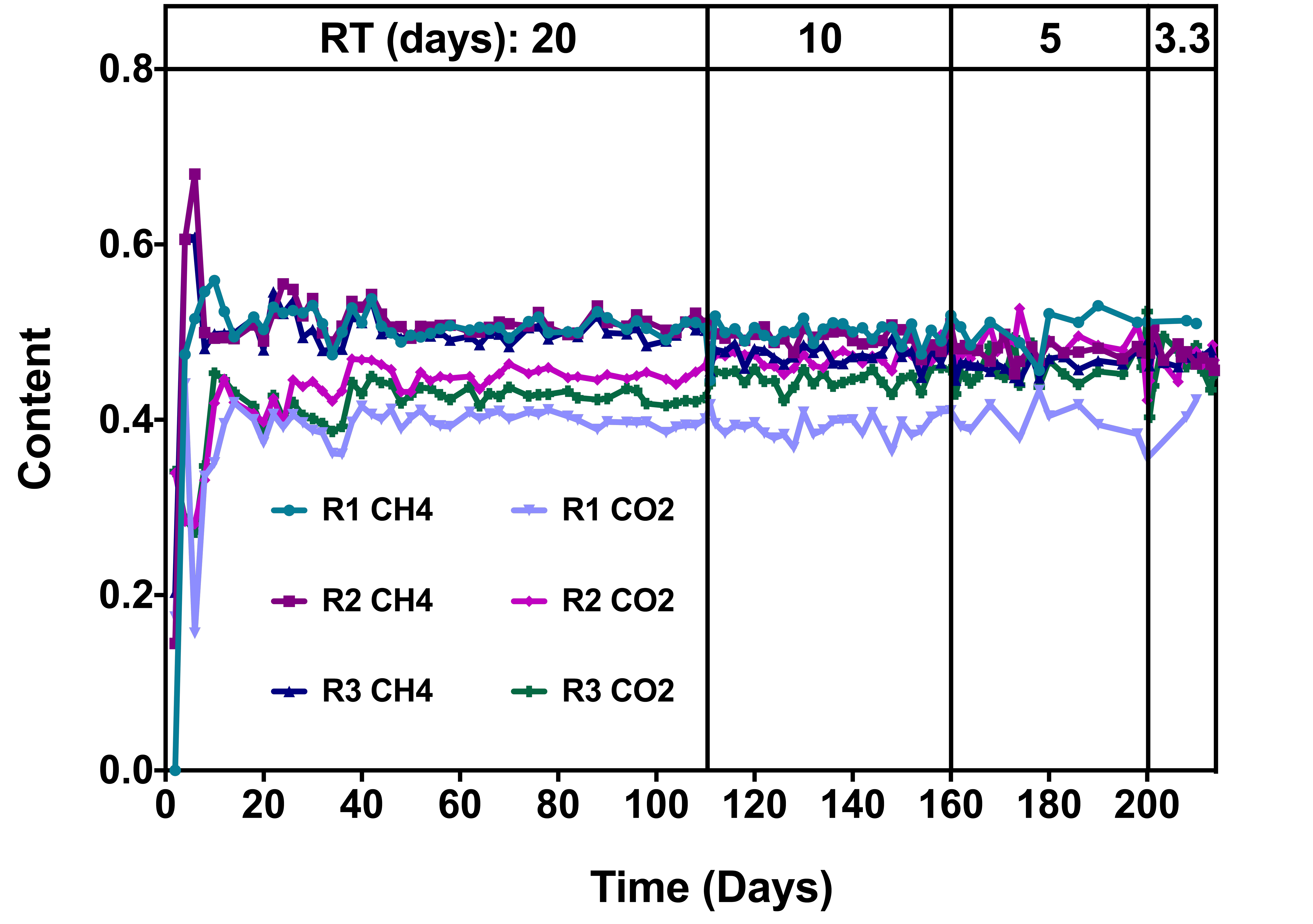

Supplement: Supplementary file 1 — Additional file 1: Figure S1. Biogas composition vs. time. R1 was the control reactor and maintained at residence time (RT) = 20 days throughout. R2 and R3 had decreasing RTs (20 days, 10 days, 5 days and 3.3 days) with each RT’s period indicated by solid black lines. Contents of CH4 and CO2 were expressed on fractional basis. [file 13068_2018_1238_MOESM1_ESM.tiff]

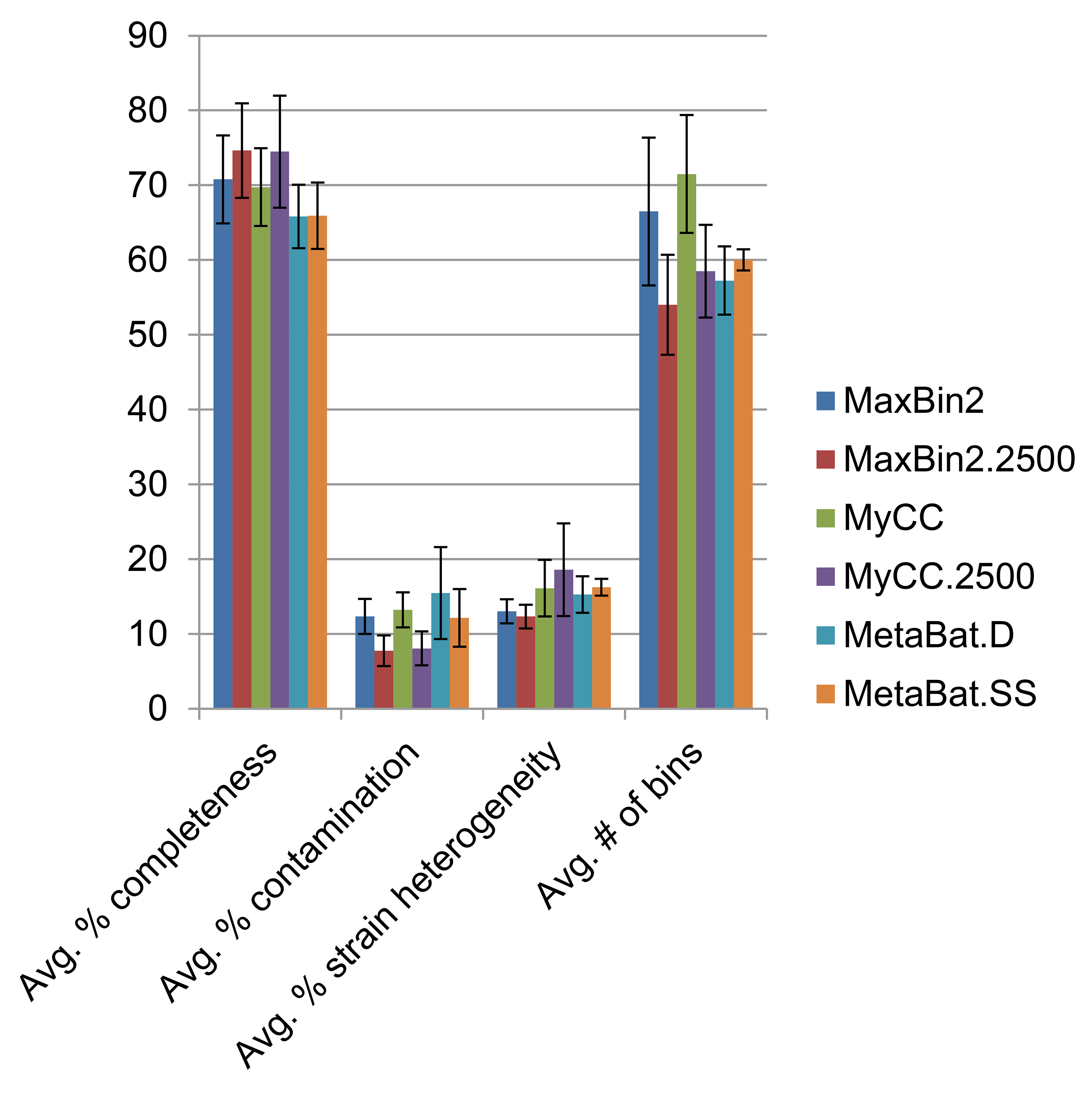

Supplement: Supplementary file 5 — Additional file 5: Figure S2. Comparison of statistics generated from four metagenomes from reactor R3 that were binned with MaxBin2, MyCC, and MetaBat programs. Default parameters were tested. Since the default minimum contig length of MetaBat was 2500 nucleotides, MaxBin and MyCC were also tested with this parameter. The “superspecific” MetaBat parameter settings were also tested. Error bars indicate the standard deviation of averaged binning statistics of the four metagenomes. Significantly different means were identified by ANOVA at an α = 0.05 for the % contamination and number of bins. However, a Tukey–Kramer post hoc test only identified a significant difference in the mean number of bins for MyCC compared to MaxBin2.2500. [file 13068_2018_1238_MOESM5_ESM.tif]

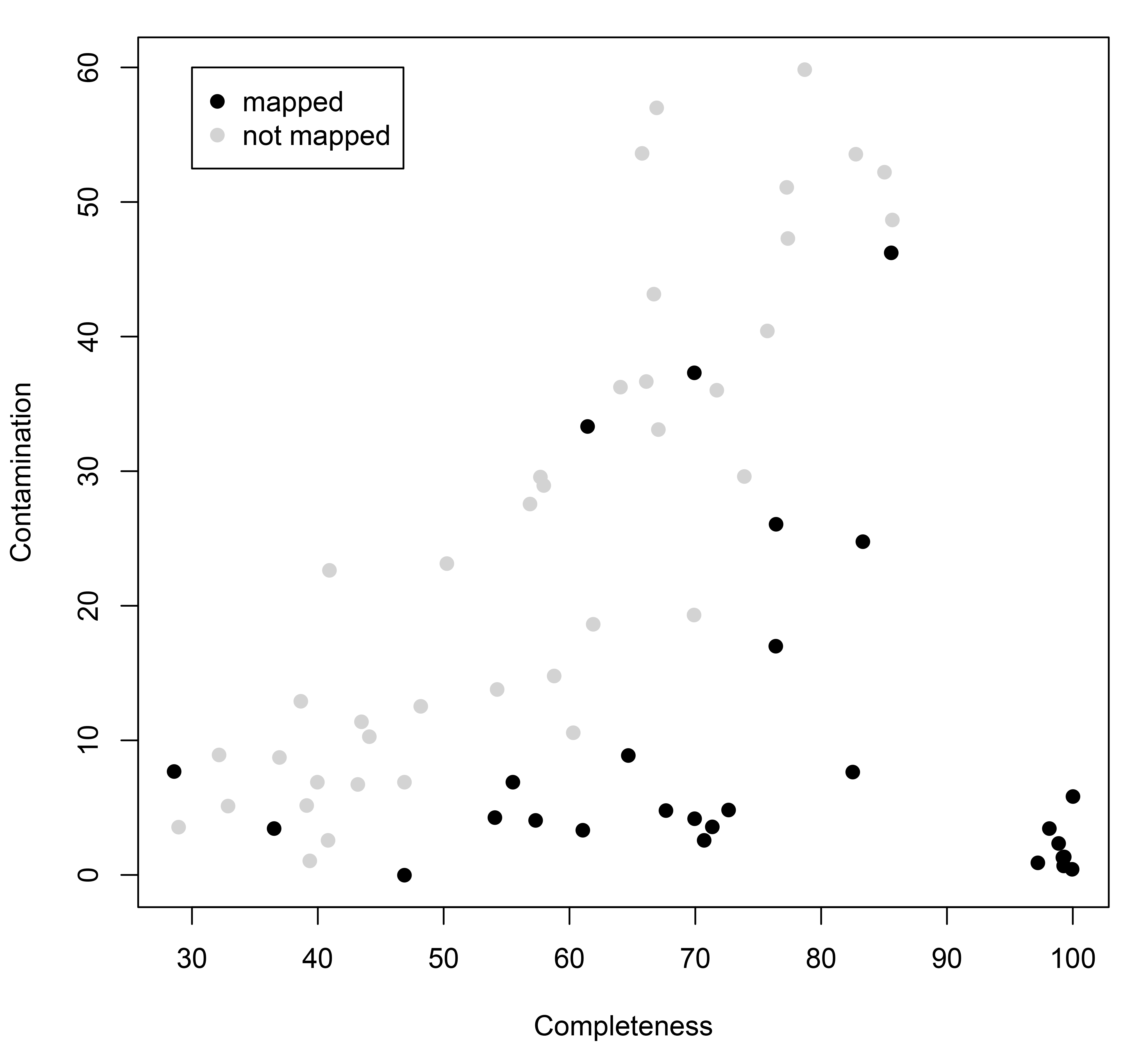

Supplement: Supplementary file 6 — Additional file 6: Figure S3. MapBin. Plot indicating the completeness and contamination distribution of bins that can or cannot be mapped. X-axis indicates completeness ratio (%) while Y-axis means contamination ratio (%). Black dots are bins that can be mapped; gray ones are bins that cannot. [file 13068_2018_1238_MOESM6_ESM.tif]

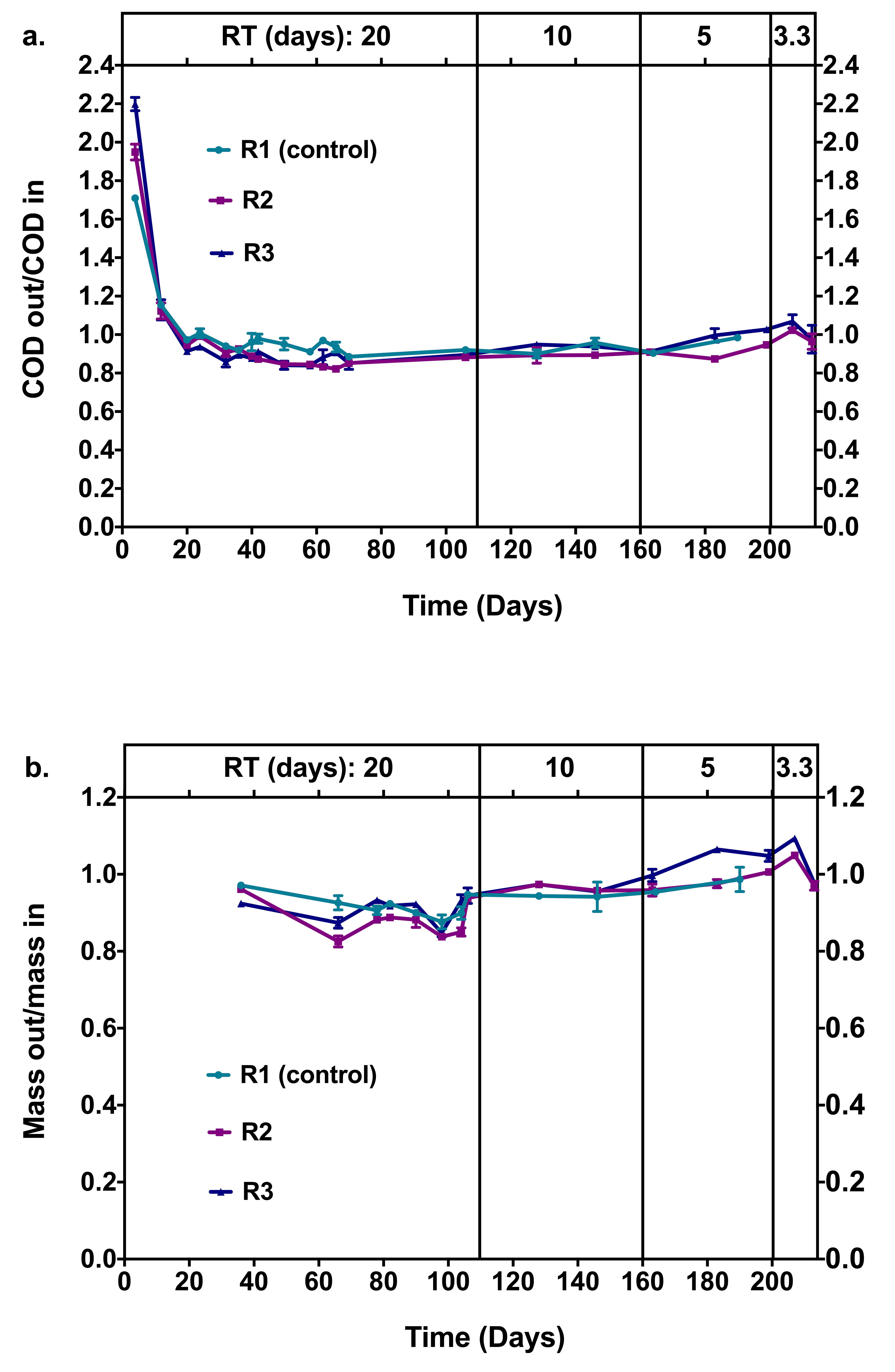

Supplement: Supplementary file 9 — Additional file 9: Figure S4. a. COD recovery vs. time; b. Mass recovery vs. time. R1 was the control reactor and maintained at residence time (RT) = 20 days throughout. R2 and R3 had decreasing RTs (20 days, 10 days, 5 days and 3.3 days) with each RT’s period indicated by solid black lines. Results were expressed as mean ± SD. [file 13068_2018_1238_MOESM9_ESM.tiff]

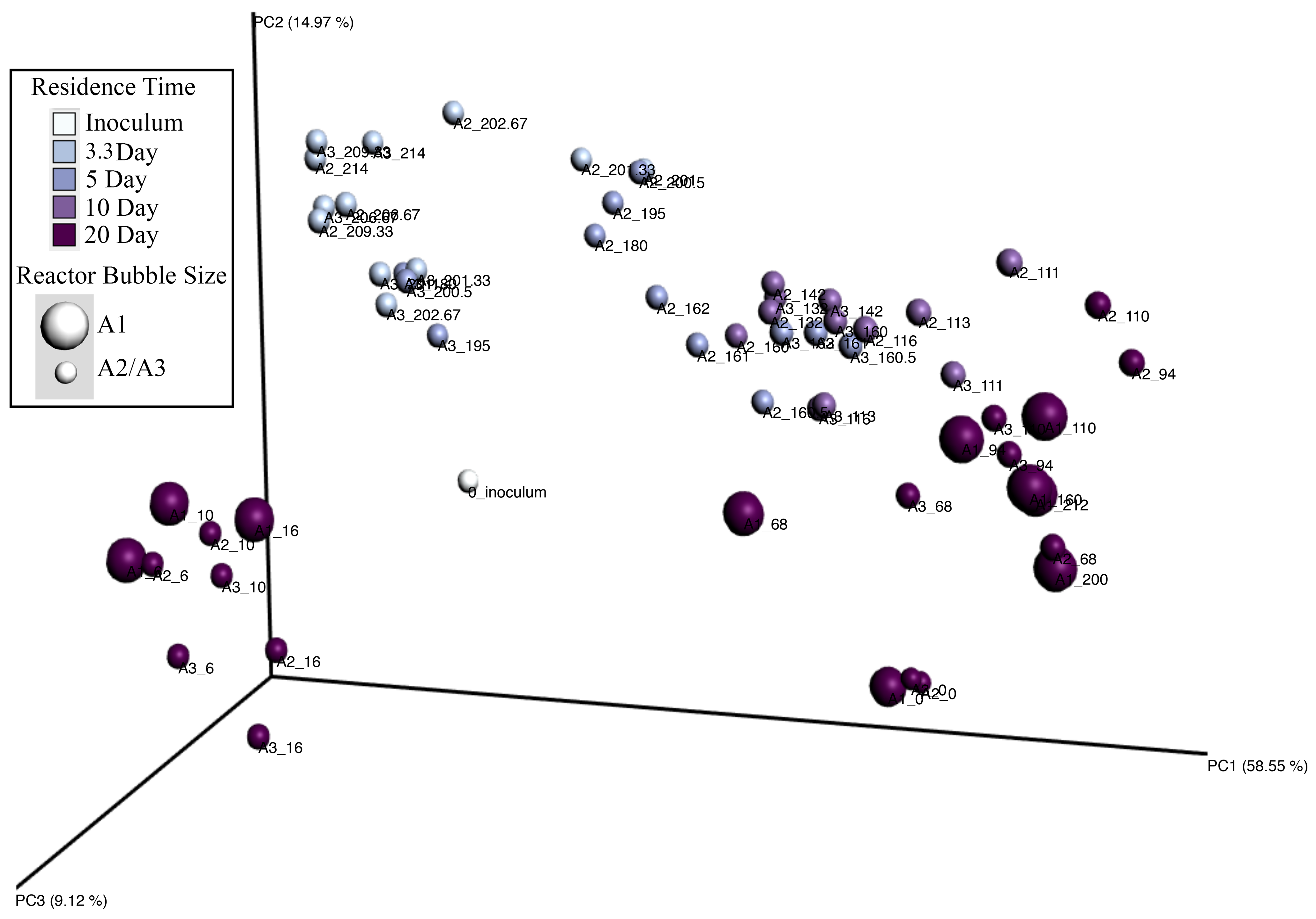

Supplement: Supplementary file 10 — Additional file 10: Figure S5. PCoA plot of sample phylogenetic distance. Phylogenetic distance of samples represented by weighted UniFrac values. Residence time (RT) is represented by different colors. Reactor 1 (A1) samples are twice the size of samples for reactors 2 (A2) and 3 (A3) reactors. Samples are labeled with [reactor]_[day]. [file 13068_2018_1238_MOESM10_ESM.tif]

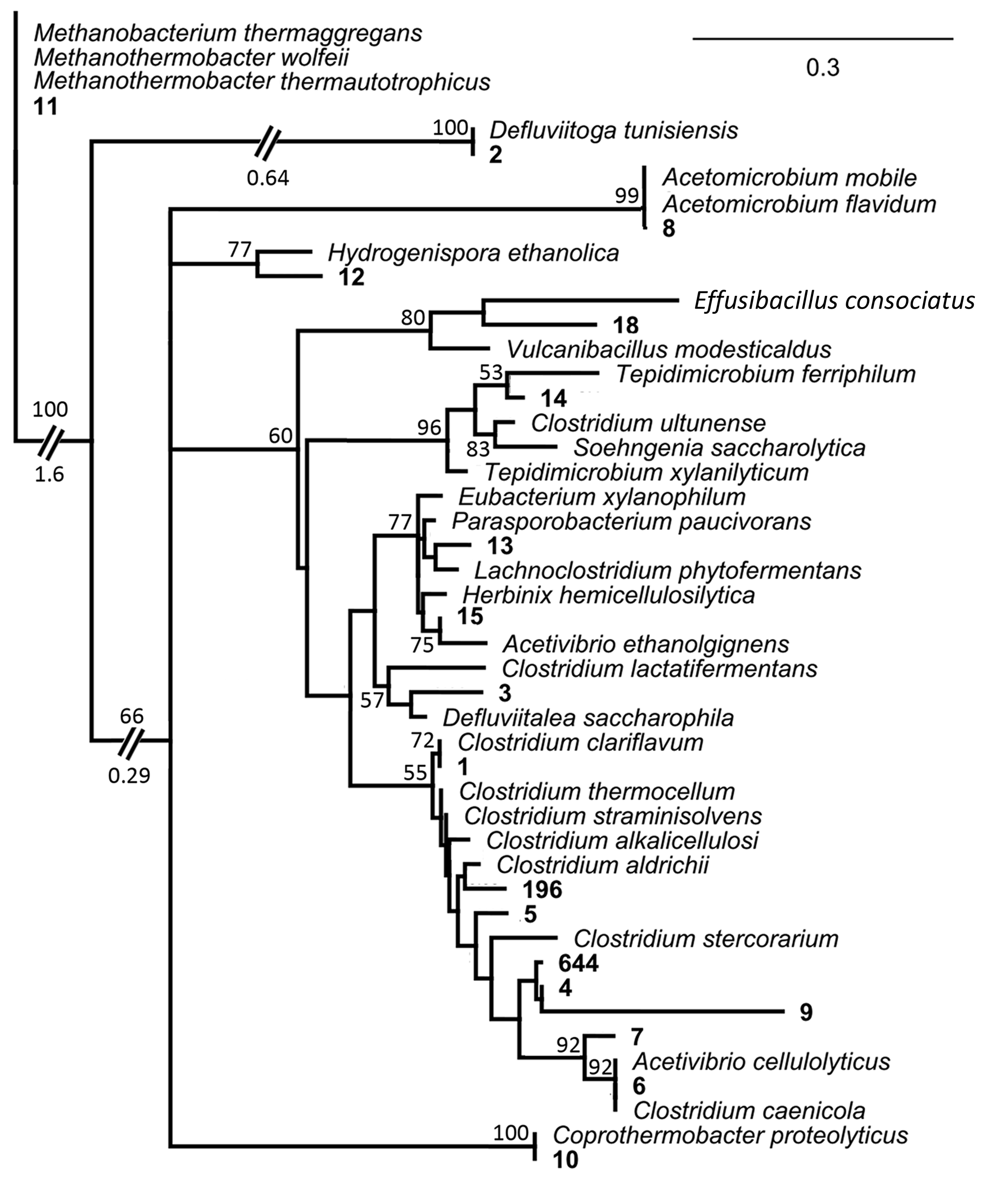

Supplement: Supplementary file 11 — Additional file 11: Figure S6. Maximum likelihood phylogenetic tree of the most abundant operational taxonomic units in the switchgrass digesters (bold numbers), which when summed together account for greater than 80% of the average relative abundance. Numbers given to OTUs only allude to the abundance of the OTUs in one of the 61 switchgrass digester samples. Numbers adjacent to branches show bootstrap supports that were greater than 50%. The scale bar and numbers below the branch-breaks indicate the number of nucleotide substitutions per position in the V4 region of the 16S rDNA gene sequences. [file 13068_2018_1238_MOESM11_ESM.tif]
