# Supplementary material for: Development and characterization of stable anaerobic thermophilic methanogenic microbiomes fermenting switchgrass at decreasing residence times
Source: Biotechnol Biofuels. 2018 Sep 6;11:243. doi: 10.1186/s13068-018-1238-1 (PMC6126044; doi:10.1186/s13068-018-1238-1)
Supplement: Supplementary file 2 — Additional file 2: Table S1. ANOVA of effects of residence time (RT) and anaerobic/aerobic sampling method on total carbohydrate solubilization. [file 13068_2018_1238_MOESM2_ESM.pdf]

Table S1 ANOVA of effects of residence time (RT) and anaerobic/aerobic sampling method on total carbohydrate solubilization.

**Factor information**					
Factor	Type	Levels	Values		
RT (days)	Fixed	4	3.3, 5.0, 10.0, 20.0		
Sampling method	Fixed	2	Aerobic, Anaerobic		
**Analysis of variance**					
Source	DF	Adj SS	Adj MS	F-Value	P-Value
RT (days)	3	0.151656	0.050552	162.08	<0.001
Sampling method	1	0.000493	0.000493	1.58	0.217
RT*Sampling method	3	0.004966	0.001655	5.31	0.004
Error	36	0.011228	0.000312		
Total	43	0.168392			

A two-way ANOVA was conducted with Minitab 17 Statistical Software (State College, PA: Minitab, Inc. (www.minitab.com)) on a sample of 44 data points to investigate the effect of residence time (RT) and sampling method on total carbohydrate solubilization. Analysis showed that RT had significant ($p<0.001$) effect on solubilization, while there was no significant difference ($p=0.217$) between aerobic sampling and anaerobic sampling method. The interaction between the effects of RT and sampling method on solubilization was also significant ($p=0.004$).
