# Supplementary material for: Development and characterization of stable anaerobic thermophilic methanogenic microbiomes fermenting switchgrass at decreasing residence times
Source: Biotechnol Biofuels. 2018 Sep 6;11:243. doi: 10.1186/s13068-018-1238-1 (PMC6126044; doi:10.1186/s13068-018-1238-1)
Supplement: Supplementary file 3 — Additional file 3: Table S2. ANOVA of effects of residence time (RT) and anaerobic/aerobic sampling method on CH4 content. [file 13068_2018_1238_MOESM3_ESM.pdf]

**Table S2** ANOVA of effects of residence time (RT) and anaerobic/aerobic sampling method on CH_4 content.					
**Factor information**					
Factor	Type	Levels	Values		
RT (days)	Fixed	4	3.3, 5.0, 10.0, 20.0		
Sampling method	Fixed	2	Aerobic, Anaerobic		
**Analysis of variance**					
Source	DF	Adj SS	Adj MS	F-Value	P-Value
RT (days)	3	0.018570	0.006190	65.35	<0.001
Sampling method	1	0.000847	0.000847	8.94	0.004
RT*Sampling method	3	0.000820	0.000273	2.89	0.041
Error	82	0.007767	0.000095		
Total	89	0.029394			

A two-way ANOVA was conducted with Minitab 17 Statistical Software (State College, PA: Minitab, Inc. (www.minitab.com)) on a sample of 90 data points to investigate the effect of residence time (RT) and sampling method on CH_4 content of biogas. Analysis showed that both RT and sampling method had significant ($p<0.001$, $p=0.004$) effect on CH_4 content. The interaction between the effects of RT and sampling method on CH_4 content was also significant ($p=0.041$).
