# Supplementary material for: Development and characterization of stable anaerobic thermophilic methanogenic microbiomes fermenting switchgrass at decreasing residence times
Source: Biotechnol Biofuels. 2018 Sep 6;11:243. doi: 10.1186/s13068-018-1238-1 (PMC6126044; doi:10.1186/s13068-018-1238-1)
Supplement: Supplementary file 4 — Additional file 4: Table S3. Steady State Data Summary. Results are expressed as mean ± SD. [file 13068_2018_1238_MOESM4_ESM.pdf]

Table S3 Steady State Data Summary. Results are expressed as mean ± SD.

**RT (days)**	Reactor 1			Reactor 2			Reactor 3		
	Mean ± SD		# of data pts	Mean ± SD		# of data pts	Mean ± SD		# of data pts
	Fractional carbohydrate solubilization								
**20**	0.712±0.019		24	0.697±0.012		7	0.72±0.024		7
**10**				0.664±0.026		7	0.644±0.014		7
**5**				0.594±0.011		6	0.568±0.008		6
**3.3**				0.542±0.024		2	0.535±0.010		2
	Biogas production rate (mL/day)								
**20**	465.3±16.8		76	416.8±18.9		25	458.2±29.9		25
**10**				851.3±65.5		20	928.8±51.1		20
**5**				1530.8±142.7		51	1694.4±117.6		51
**3.3**				2422.8±283.1		10	2324.3±141.1		10
	Biogas composition								
	CH_4 (%)	CO_2 (%)		CH_4 (%)	CO_2 (%)		CH_4 (%)	CO_2 (%)	
**20**	50.3±1.5	39.7±1.4	38	50.9±0.9	45.1±0.7	20	49.9±0.9	42.6±0.7	20
**10**				49.1±1.3	47.2±1.3	10	47.5±1.3	44.8±1.1	10
**5**				47.8±0.6	47.6±2.4	11	46.9±1.1	46.0±3.1	11
**3.3**				46.4±0.6	47.0±1.0	4	47.1±0.7	44.9±1.0	4
	Volatile fatty acids (g/L)								
	Propionic	Acetic		Propionic	Acetic		Propionic	Acetic	
**20**	0.017±0.030	0	31	0	0	7	0	0	7
**10**				0	0	7	0.224±0.033	0	7
**5**				0.321±0.060	0	6	0.335±0.056	0	6
**3.3**				0.285±0.020	0	2	0.314±0.047	0	2
	pH[α]								
**20**	6.72±0.05		487	6.45±0.02		159	6.43±0.04		159
**10**				6.40±0.02		64	6.36±0.03		64
**5**				6.18±0.04		81	6.25±0.02		81
**3.3**				6.04±0.03		10	6.15±0.02		10
	Electron balance analysis								
**20**	93.2±3.6%		8	84.7±2.6%		4	88.4±2.5%		4
**10**				89.3%		1[β]	93.8%		1
**5**				91.0±5.2%		2	101.1±2.2%		2
**3.3**				96.4%		1	97.6%		1
	Mass balance								
**20**	92.8±3.1%		11	87.2±3.8%		7	91.0±3.6%		7
**10**				95.8%		1	95.6%		1
**5**				99.1±2.1%		2	105.7±1.2%		2
**3.3**				97.1%		1	97.8%		1

[α] The dataset used was subsampled from the original pH dataset, which had 20 data points per hour and in total over 60,000 points. When subsampling, for every 100 points only one point was kept and the rest was eliminated. This led to 3-4 data points per day in the final pH dataset presented.

[β] If only sample from one time point were analyzed, then the standard deviation was not shown since it’s only from analytical replicates.
