# Supplementary material for: Development and characterization of stable anaerobic thermophilic methanogenic microbiomes fermenting switchgrass at decreasing residence times
Source: Biotechnol Biofuels. 2018 Sep 6;11:243. doi: 10.1186/s13068-018-1238-1 (PMC6126044; doi:10.1186/s13068-018-1238-1)
Supplement: Supplementary file 8 — Additional file 8: Table S5. Reactor operating length and steady state length at each residence time (RT). R1 was the control reactor always running at RT = 20 days and R2 & R3 had decreasing RTs. [file 13068_2018_1238_MOESM8_ESM.pdf]

Table S5 Reactor operating length and steady state length at each residence time (RT). R1 was the control reactor always running at RT = 20 days and R2 & R3 had decreasing RTs.

**RT (days)**	Total				Steady state			
	Length (days)		Time period		Length (days)		Time period	
	R1	R2&R3	R1	R2&R3	R1	R2&R3	R1	R2&R3
**20**	212	110	1st – 212th	1st – 110th	152	50	61th – 214th	61th – 110th
**10**		50		111st – 160th		20		141th – 160th
**5**		40.5		160.5th – 200.5th		25.5		175.5th – 200.5th
**3.3**		13.3		200.67th –213.67th		3.3		210.67th – 213.67th
